# Nutrition, stress, and healthcare use during pregnancy are associated with low birth weight: evidence from a case–control study in West Ethiopia

**DOI:** 10.3389/fpubh.2023.1213291

**Published:** 2023-10-20

**Authors:** Bikila Regassa Feyisa, Yosef Mulatu, Firehiwot Fentahun, Bayise Biru, Evan Atlantis

**Affiliations:** ^1^Department of Public Health, Institute of Health Sciences, Wallaga University, Nekemte, Ethiopia; ^2^Department of Epidemiology, Faculty of Public Health, Jimma University, Jimma, Ethiopia; ^3^World Vision Ethiopia-Gulliso, Addis Ababa, Ethiopia; ^4^Department of Human Nutrition and Dietetics, Faculty of Public Health, Jimma University, Jimma, Ethiopia; ^5^School of Health Sciences, Western Sydney University, Penrith, NSW, Australia; ^6^Translational Health Research Institute, Western Sydney University, Sydney, NSW, Australia

**Keywords:** low birth weight, case–control, maternal factors, stress, West Ethiopia

## Abstract

**Background:**

Low Birth Weight (LBW) remains a major public health concern globally, especially in lower and middle-income countries. In Ethiopia in general and in the study area in particular there is limited evidence regarding maternal factors associated with low birth weight. This study aimed to identify the maternal factors associated with low birth weight among neonates born in public health facilities in the west Wollega zone, West Ethiopia.

**Methods:**

We used a case–control study design and selected participant mothers with a newborn using the delivery database from three public hospitals and five health centers, between March 2022 and April 2022. Cases were identified using a newborn birth weight of <2,500 grams. Controls were identified using a newborn birth weight of ≥2,500 grams. An interviewer-administered structured questionnaire and chart review were used for data collection. Model fitness was assessed by Hosmer and Lemenshow goodness-of-fit test, including multi-collinearity checks. Candidate predictor variables were selected (using a value of *p* <0.25 in bivariable logistic regression models) for multivariable logistic regression to quantify the association between independent variables and LBW, expressed using Odds Ratios (OR) with a 95% Confidence Interval (CI). Mean and Standard Deviation (SD), all such values.

**Results:**

A total of 324 mothers with their newborns (81 cases and 243 controls) were eligible for inclusion. The mean age of participants was 27.9 years (SD 6.4) in cases and 25 years (SD 3.9) in controls. Mean birth weight was 2,128 grams (SD 1,697) in cases and 2,988 grams (SD 378) in controls. In multivariable logistic regression analysis, lack of nutritional counseling (OR = 2.4; 95%CI: 1.24–4.72), maternal middle upper arm circumference of <23 cm (OR = 3.1; 95%CI: 1.64–5.91), maternal stress during pregnancy (OR = 2.8; 95% CI:1.23–6.36), and antenatal follow up less than four visits (OR = 2.8; 95% CI: 1.12–6.82) were independently associated with LBW.

**Conclusion:**

In this study, lack of nutritional counseling, maternal undernutrition, maternal stress during pregnancy, and antenatal follow-up visits less than recommended were associated with LBW. Special attention should be given to promoting antenatal care and counseling mothers on nutrition and relaxation to prevent stress during pregnancy.

## Introduction

Low birth weight (LBW) remains a major public health concern globally, especially in Lower and Middle Income Countries (LMIC) (1). The World Health Organization (WHO) LBW as a weight at birth of less than 2,500 grams (or 5.5 pounds) (1). The weight of a baby at term depends on its gestational age and the rate of fetal growth in the uterus. An infant’s birth weight is the first weight measured after birth, ideally within the first few hours after birth, before considerable postnatal weight loss occurs (2). Globally, it is estimated that 15–20 percent of all births are LBW, resulting in over 20 million new cases born each year and 60–80% of newborn deaths (3, 4). It is estimated that LMIC accounts for more than 90% of LBW cases worldwide, with 72 and 22% born in Asia and Africa, respectively (5).

Evidence shows that babies with a LBW are associated with adverse physiological, psychological, and functional health and development into adulthood (6, 7). For instance, LBW babies are more likely to have stunted growth and poor cognitive development in their early years (8). As a result, LBW results in reduced productivity in education, economic, and other activities. The risk of developing cardiovascular diseases, diabetes and hypertension is higher later in their life (9, 10). It also contributes to the inter-generational cycle of poverty, malnutrition, and diseases often experienced in LMIC (9, 11). Furthermore, LBW has economic consequences not only the family members but often the entire community because of increased medical costs, time spent in hospitals, and time away from work. The identification of potentially modifiable risk factors for LBW is required for developing effective prevention interventions in LMIC.

In LMIC, LBW is common among mothers with further socio-economic disadvantage and poor access to health care, associated with periconceptual stressors (12). These inequalities are associated with maternal risk factors for LBW including inadequate nutrition, restricted blood supply to the uterus as a result of stress, specific and non-specific illnesses, and complications during pregnancy (13, 14). For instance, lack of support for stress management during pregnancy is exacerbated by socio-economic disadvantage and poor health behaviors (9, 15). If neglected, the health and well-being of expectant mothers is likely to exacerbate the risk of LBW for their newborns (16, 17).

Published literatures indicated that there are evidences showing LBW is common and plays a substantial role in neonatal, infant, and under-five mortality (13). It is increasingly being used to predict prenatal mortality and morbidity, particularly in Sub-Saharan African countries, including Ethiopia (13). In Ethiopia, the mini demographic health survey in 2019 reported a neonatal mortality rate of 30 per 1,000 live births (17, 18). Reducing the rate of LBW is vital for reducing child mortality (19), which is critical to the future development of LMIC. Nearly 3 million of these deaths occur within the first four weeks of life, contributing considerably to neonatal mortality rates that have nearly doubled in recent years (20). Each year, Ethiopia loses approximately 27,587 neonates due to LBW, accounting for 4.5% of total neonatal mortality (19). The prevalence of LBW is as high as 18% in Ethiopia, which indicates that almost one in five neonates weighs less than the normal range (21). Furthermore, those with LBW but survive the neonatal period are more likely to develop chronic disease in adulthood, such as cardiovascular diseases and diabetes (1, 9).

A range of maternal risk factors for LBW in LMIC have been implicated but are poorly understood. Research shows that high maternal blood pressure during pregnancy, maternal age, the mother’s educational status, insufficient gestational weight gain, not taking iron and folic acid during pregnancy, maternal Middle Upper Arm Circumference (MUAC) of <23 cm reflecting nutritional status during famine (22, 23), lack of nutritional counseling during pregnancy, anemia, incomplete antenatal care, preterm birth, lack of additional food intake during pregnancy, and a history of intimate partner violence are among the risk factors for LBW (11, 24, 25). However, the strength and independence of these associations are not well-defined. This is because, most of the pocket studies were cross-sectional studies, where the temporal relationship of the predictor variables and outcome variable is less identified, and also no comparison group identified in cross-sectional studies. Therefore, the current study aims to identify the maternal risk factors associated with LBW in Ethiopia.

## Materials and methods

We present this paper according to the Journal’s formatting requirements and STROBE guidelines for reporting case–control studies.

### Study setting, design, and period

This study was conducted in the selected health facilities in the west Wollega zone. West Wollega is found in Oromia regional state, which is located 441 km away from Addis Ababa to the west. There were five public hospitals, two non-governmental organization hospitals, and 67 health centers providing health services, including delivery services. This study was conducted in three hospitals (Gimbi general hospital, Nedjo general hospital, and Mendi primary hospital), and five health centers (Bila health center, Guliso health center, Nedjo health center, Muklami health center, and Dongoro health center), which were selected by lottery method. First, all the hospitals and health centers found in the zone were listed. We identified separately the hospitals and health centers based on the case load, and geographical variations by which they provide the services. Accordinlgy, we selected a total of eight health facilities using lottery method. An institutional-based unmatched case–control study was conducted from March to April 2022.

### Study population

The source population was all mothers who gave birth in the selected public health facilities in the west Wollega zone. All mothers who gave singleton live births in the selected public health facilities in the West Wollega zone during the study period who met eligibility criteria were selected as the study population.

### Cases

Those live singleton babies with birth weight less than 2,500 grams.

### Controls

Those live singleton babies with birth weight greater than or equal to 2,500 grams.

### Eligibility criteria

All mothers who gave live births in the selected public health facilities in the study area and who gave informed consent and who were able to give information were included in the study, while maternal cards having incomplete information, multiple births, mothers who lose infants after birth, preterm neonates and mothers or mother in the critical medical conditions were excluded from the study.

### Sample size determination and sampling procedure

The sample size was calculated using Epi Info version 7 software for an unmatched case–control study using the following assumptions: 95% confidence level (zα/2 = 1.96), power (Zβ = 80%), case to control ratio 1:3 (r = 3), odds ratio to be detected 2.84 and 10.8% (25) of the control group to be exposed. After adding a 10% non-response rate, the final sample size was 324 (81 cases and 243 controls).

We used the last two months of the consecutive year of delivery performance to proportionally allocate the sample size to each health facility, which was 4,289 deliveries among the selected health facilities in 2022. Babies of mothers who delivered during the study periods were measured using the calibrated scale within 30 min of delivery. Cases (birth weight less than 2,500 grams) were included in the study and three consecutive mothers in the controls (birth weight greater than or equal to 2,500 grams) were interviewed. Delivery registers and Antenatal Care (ANC) cards were reviewed in addition to the interview-administered questionnaires.

### Study variables

The outcome variable was low birth weight. The exposure variables were maternal socio-demographic variables (age, ethnicity, maternal educational status, marital status, place of residence, maternal employment, and household income), maternal obstetric factors: pregnancy-related complication, parity, gravidity, pregnancy type, previous history of LBW (frequency of ANC follow up, gestational weight gain, anemia, and hypertension), maternal psycho-social (Stress during pregnancy), and maternal nutrition and health promotion factor (nutritional counseling, iron supplementation, and maternal MUAC).

### Operational definitions

- Low birth weight is weight at birth of less than 2,500 grams (1).
- Maternal stress is feeling overwhelmed during pregnancy resulting from changes in body structure and composition and as well as emotions (26).

### Data collection tools, data collectors, and the procedures

The questionnaires were adapted from the Ethiopian Demographic Health Survey (EDHS) and previous studies (25, 27, 28). These questionnaires have five components: socio-demographics, obstetrics, current pregnancy, nutritional factors, and psychosocial factors. The data was collected by interviewing the mothers after the mother was calmed using a structured and pretested questionnaire. Maternal MUAC was measured after removing her clothes using a standard WHO MUAC measuring tape.

Maternal stress was assessed based on validated ten standard Perceived Stress Scales (PSS) (29). First, negatively worded questions 4, 5, 7, and 8 were reversed to the positive ones. Then, the total individual PSS score was added up on a linear scale and ranged from 0–40 with a higher score indicating higher perceived stress. Accordingly, the maternal stress scale score was categorized as low stress (0–13), moderate stress (14–26), and high stress (27–40) score. Maternal hemoglobin level was extracted from ANC registration of respective health facilities to determine the status of maternal anemia. Those whose hemoglobin level was less than 11.00 g/dL were considered as having anemia, while those who had a hemoglobin level of greater or equal to 11 g/dL were considered normal. The weight of the neonates was measured using a balanced Seca scale (Germany) to the nearest 0.01 kg.

Eight experienced BSc midwives were engaged in data collection, supervised by two senior BSc midwives. The data was collected each time a low-birth-weight baby is delivered until the required sample size was obtained. The data collectors visited the post-natal, delivery room, and neonatal wards three times a day (morning, afternoon, and evening) to identify the eligible study participants. Also, the staff on duty at the post-natal room and neonatal units alerted the data collectors each time a delivery, which meets the case definition and inclusion criteria indicated. In addition to the structured interview administered questionnaire, the antenatal record book was reviewed as well as maternal MUAC was measured and recorded.

### Data quality control

A structured interview questionnaire was prepared in English language and then translated into Afaan Oromoo (the working language of Oromia regional state) for data collection and then retranslated back into English by experts in both languages to keep the language consistent. Two days of training were given to data collectors and supervisors to familiarize them with data collection tools. A pre-test was done on 5% of the sample sizes at Gimbi health center before starting the actual data collection, and necessary modifications were made regarding the order of the questioners, the wordings, and its content. Informed written consent was taken before the start of data collection from each participant. The data were collected within 24 h of delivery, and the response to questionnaires was filled immediately. Before taking weight, towels were taken off and the rechecking of the function of the weight scale between measurements was confirmed using a standard scale. After each measurement, the scale was calibrated to zero. The data were collected within 24 h of delivery. The confidentiality of the collected data is ensured.

## Data processing and analysis

The collected data was coded and checked for completeness and consistency. Identified errors during this time were adjusted after a review of the original data using the code numbers. Data were entered into EpiData software version 3 and exported to the Statistical Package for Social Science (SPSS) software version 26 for cleaning and analysis.

Frequency distribution and summary statistics such as mean and standard deviation were computed for cases and control groups. Model fitness was assessed by using the Hosmer and Lemeshow goodness of fit test (*p* = 0.56), showing the model was a good fit for the data. The bivariable regression model was carried out, and variables having a value of p less than or equal to 0.25 were entered into the multivariable logistic regression model. Multi-collinearity was checked using Variance Inflation Factor (VIF), with a maximum of 1.9, indicating no multicollinearity. The multivariable logistic regression model was built using the enter method in the final model. Finally, a value of p less than 0.05 with its 95% Confidence Interval (CI) was used to declare the statistical significance.

## Results

### Socio-demographic characteristics of the respondents

A total of 324 mothers who gave singleton birth were approached, and all were included in the analysis as 81 neonates who had LBW with their mothers (cases) and 243 neonates who had not LBW with their mothers (controls). The mean age of the mothers in cases was 27.9 years (SD 6.4), and the age of mothers in controls was 25 years (SD 3.9). The mean birth weight was 2,200 grams (IQR 300) among cases, and 2,900 grams (IQR 500) among controls. Forty-six (56.8%) of case mothers and 134 (55.1%) control mothers lived in rural areas (Table 1).

**Table 1:** Frequency distribution of socio-demographic factors among women delivered in public health facilities of west wollega zone, Ethiopia.

variables and categories		Birth weight, *n* (%)		*p* value
		Cases (81)	Controls (243)	
Age category	<20	14 (17.3)	27 (11.1)	0.571^a^
	20–34	48 (59.3)	210 (86.4)	
	≥35	19 (23.5)	6 (2.5)	
Marital status	Married	63 (77.8)	239 (98.4)	0.691^a^
	Single	8 (9.9)	1 (0.4)	
	Divorced	10 (12.3)	3 (1.2)	
Residence	Urban	35 (43.2)	109 (44.9)	0.550^b^
	Rural	46 (56.8)	134 (55.1)	
Educational status	Employed	9 (11.1)	62 (21.9)	0.200^**a**^
	Daily laborer	32 (39.5)	68 (30.9)	
	Merchant	9 (11.1)	48 (17.6)	
	House wife	21 (25.9)	57 (24.1)	
	Other*	10 (12.3)	8 (5.6)	

^*^student, tailor, hair dresser.

a pearson chi-square.

b Fischer’s Exact Test.

### Obstetric, nutritional, and psycho-social characteristics

Three fourth of the mothers (76.5%) had a planned pregnancy and 19 (23.5%) experienced an unwanted pregnancy. Thirty percent of women among cases and 20% among control had suffered pregnancy-related problems. Anemia, gestational hypertension, chronic hyperglycemia, and chronic hypertension were the most common pregnancy-related problems among the cases, with 15 (18.5%), 14 (17.8%), 7 (8.6%), and 14 (17.3%), respectively. Anemia, pregnancy induced hypertension, and chronic hypertension were found in 17 (7%), 14 (5.8%), and 8 (3.3%) of the control women, respectively. Most of the women among cases (51.8%) gave birth vaginally followed by Caesarian Section (CS) and assisted instrumental containing 34 (37.8%) and 4 (4.4%) respectively. More than one forth cases 21 (36.2) and 40 (23.3%) of controls bear children in less than two years’ period. Nearly half of cases 32 (45.7%) compared with 41 (18.5%) of controls had received antenatal care of <4 visits. Only one fourth of the cases and 146 (60.1%) control groups had nutritional counseling during pregnancy. Sixty percent of cases and 64 (26.3%) control mothers had MUAC less than 23 cm (Table 2).

**Table 2:** Obstetrics, nutritional and psycho-social characteristics of mothers who gave birth at public health facilities in west Wollega zone, Ethiopia.

variables and categories		Birth weight, *n* (%)		*p* value
		Cases (81)	Controls (243)	
Nutritional counseling during pregnancy	Yes	23 (28.4)	146 (60.1)	0.001^**b**^
	No	58 (71.6)	97 (39.9)	
Additional meals during pregnancy	Yes	35 (43.2)	137 (56.4)	0.027^**b**^
	No	46 (56.8)	106 (43.6)	
Take iron during pregnancy	Yes	67 (82.7)	222 (91.4)	0.028^**b**^
	No	14 (17.3)	106 (8.6)	
Number of Iron tab received	<100	41 (62.9)	100 (45.5)	0.008^**b**^
	≥100	26 (37.1)	123 (54.5)	
Maternal MUAC	<23 cm	49 (60.5)	64 (26.3)	0.001^**b**^
	≥ 23 cm	32 (39.5)	179 (73.7)	
Gestational weight gain	<12 kg	37 (45.7)	96 (39.5)	0.196^**b**^
	≥ 12 kg	44 (54.3)	147 (60.5)	
ANC visit	Yes	67 (82.7)	224 (92.2)	0.044^**b**^
	No	14 (17.3)	19 (7.8)	
Frequency of ANC	<4	32 (45.7)	41 (18.5)	0.001^**b**^
	≥4	38 (54.3)	181 (81.5)	
Maternal stress level	Low	22 (27.2)	110 (45.3)	0.001^**a**^
	Moderate	24 (29.6)	97 (39.9)	
	High	35 (43.2)	36 (14.8)	

ANC, Antenatal Care; MUAC, Mid Upper Arm Circumference.

a pearson chi-square.

b Fischer’s Exact Test.

**Figure 1:**
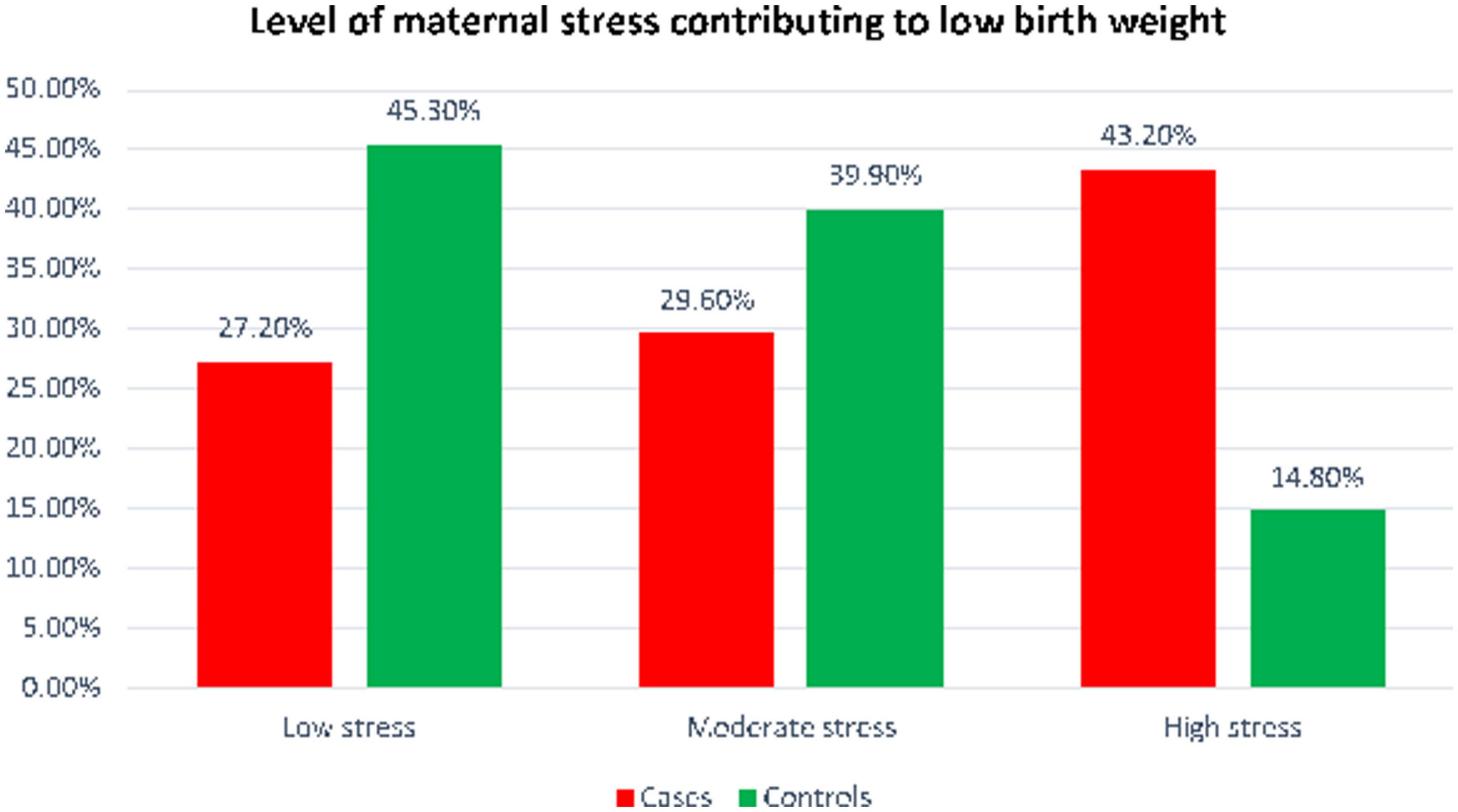
Level of maternal stress contributing to low birth weight in west Wollega zone, Ethiopia.

More than half of the women in control group and 35 (35.8%) of case group women had additional meal during pregnancy. Forty-three percent of women were subjected to stress during the current pregnancy while 36 (14.8%) women were exposed to maternal stress among control group. Nearly two in five women who had been exposed to high stress during pregnancy delivered LBW neonates (Figure 1).

### Maternal risk factors contributing to low birth weight

Maternal education, nutritional counseling, history of antenatal follow up less than 4 visits, number of iron tablet received, maternal MUAC, Iron tablet received, maternal stress, health problem during pregnancy, maternal anemia, pregnancy induced hypertension were candidate variables for the multivariable logistic regression analysis. There was no difference in birth weight among the health centers and hospitals.

In multivariable logistic regression analysis, the odds of low birth weight among women who did not get nutritional counseling during pregnancy was 2.4 times higher when compared to their counter parts (AOR = 2.4; 95% CI: 1.24–4.72). Antenatal care less than 4 visit was another predictor of low birth weight. According to the present study the likely hood of having low birth weight infants was about three times higher among women who received antenatal care less than 4 visits in compared to their counter parts (AOR = 2.8; 95% CI:1.12–6.82). Similarly, mothers who had MUAC less than 23 cm were about three times more likely to produce low birth weight infants when compared to their counter parts (AOR = 3.1; 95% CI: 1.64–5.91). Mothers who were subjected to severe stress during pregnancy had about three times higher odds of having low birth weight infants compared to their counter parts (AOR = 2.8; 95% CI: 1.23–6.36) (Table 3).

**Table 3:** Predictors of low birth weight among mothers who gave birth at public health facilities in West Wollega Ethiopia.

Variables and categories	Birth weight, *n* (%)		95% CI	
	Cases (81)	Controls (243)	COR	AOR
**Nutritional counseling**				
Yes	23 (28.4)	146 (60.1)	1	1
No	58 (71.6)	97 (39.9)	3.79 (2.19–6.56)	2.4 (1.24–4.72)*
**Educational status**				
Illiterate	7 (8.6%)	20 (8.2%)	1.93(0.67–5.55)	1.6(0.40–6.83)
primary	15 (18.5%)	62 (25.5%)	1.3(0.57–3.01)	0.83(0.29–2.41)
secondary	47 (58%)	95 (39.1%)	2.7 (1.34–5.52)	2.2(0.94–5.31)
Diploma and above	12 (14.8%)	66 (27.2%)	1	1
**Maternal MUAC**				
<23 cm	49 (60.5%)	64 (26.3%)	4 (2.28–7.27)	3.1 (1.64–5.91) *
≥23 cm	32 (39.5%)	179 (73.7%)	1	1
**Maternal stress score**				
Low stress	22 (27.2%)	110 (45.3%)	1	1
Moderate stress	24 (29.6%)	97 (39.9%)	1.2(0.653–2.35)	0.86(0.38–1.80)
High stress	35 (43.2%)	36 (14.8)	4.8 (2.53–9.34)	2.8 (1.23–6.36) *
**Pregnancy-induced HTN**				
Yes	14 (17.3)	14 (5.8)	3.4 (1.55–7.53)	1.04(0.46–4.98)
No	71 (82.7)	229 (94.2)	1	1
**Received iron tablet**				
<100 tablets	41 (62.9)	100 (45.5)	2 (1.17–3.53)	1.4(0.76–2.77)
≥100 tablets	26 (37.1)	123 (54.5)	1	1
**Frequency of ANC**				
< 4Visit	32 (45.7)	41 (18.5)	3.7 (2.09–6.64)	2.8 (1.12–6.82) *
≥4 Visit	38 (54.3)	181 (81.5)	1	1
**Presence of anemia**				
Yes	15 (18.5)	17 (7)	3 (1.43–6.37)	2.4(0.74–7.96)
No	67 (81.5)	226 (93)	1	1
**Other health problem**				
Yes	20 (24.7)	32 (13.2)	2.16 (1.89–5.86)	1(0.38–2.90)
No	61 (75.3)	211 (86.8)	1	1

ANC, Antenatal Care; AOR, Adjusted Odds Ratio; COR, Crude Odds Ratio; HTN, Hypertension MUAC, Mid Upper Arm Circumference; *p < 0.05.

## Discussion

Low birth weight is still significant in Ethiopia. The present study tried to assess maternal risk factors associated with low birth weight among women who gave birth at public health facilities of west Wollega zone, Ethiopia. The findings of the study identified that nutritional counseling during pregnancy, frequency of antenatal care, maternal under nutrition, and maternal stress during pregnancy were found to independently predicted low birth weight. All of the identified risk factors can be averted through integrated and focused maternal health care services before, during and after pregnancy.

One of the risk factors for the LBW in this study was lack of nutritional counseling during pregnancy. Women who did not receive nutritional counseling during pregnancy more than twice to give low birth weight babies when compared to their counter parts. This finding was in line with the study from Dire dawa town, Ethiopia (30), North shoa (31), Gamo Gofa (32), and Hawassa university comprehensive specialized hospital (33). This could be due to the fact that nutritional counseling may assist women improving their feeding behavior or women’s dietary practice, and thus their nutritional status. Counselled women were more likely to have appropriate dietary practice than their counterparts. Taking an adequate amount and a good quality diet is a direct determinant of gestational weight gain. Receiving dietary counseling and taking an adequate and balanced diet had a positive impact on the mothers as well the fetus’s weight during pregnancy. Moreover, healthy and optimal intrauterine fetal growth relies heavily on maternal nutrient status (34). According to the systematic review and meta-analysis of five cohort studies, the birth weight of the offspring was significantly reduced in those who had higher exposure to acrylamide when compared to those who had lower exposure to it (35). This is because, maternal acrylamide consumption is positively related with umbilical cord estradiol levels, which may influence children’s growth (36).

This study shows, the lower the MUAC of the mother less than 23 cm, the higher the chance of LBW of the fetus. This finding is consistent with the findings of study done in Ethiopia and Malaysia (37–39). Even though either acute or chronic maternal malnutrition has direct effect on the birth weight of a baby, acute maternal malnutrition has more pronounced effect. This is due to the fetal development in the uterus is strongly influenced by mother nutritional status (9). When mothers are nutritionally deficient, the fetus’s intrauterine growth is inhibited, resulting in LBW (40). Nutritional status was examined in other research as additional meal consumption and anemia throughout pregnancy, and both characteristics were found to be predictors of low birth weight (38, 40). But in the present study, the presence of anemia did not show significant association with low birth weight.

Low birth weight has been linked to moderate to high levels of stress among pregnant women. According to the present study, mothers who were subjected to severe stress during pregnancy had about three times higher odds of having low birth weight infants compared to their counterparts. This result is supported by study conducted in Ghana and China (7, 26). This could be because blood supply to the placenta decreases during times of stress. Stress increases in cortisol, norepinephrine and inflammation which affect the fetal environment and have implications for maternal and infant health. Prenatal stress exposure is negatively associated with fetal physical development. Exposure to prenatal maternal cortisol appears to be an important factor linking gestational stress to LBW and stunting. Elevating free circulating cortisol may pass through placenta, prevent fetal growth rate and reduce birth weight of the prenatally stressed offspring (26). In response to stress, blood flow to the uterus is restricted and the fetus receives fewer nutrients, which might result in LBW (41).

The odds of LBW among newborn from mother who had antenatal care less than 4 visits were 2.8 times higher as compared to their counter parts. This result is in agreement with the study conducted in Iran and Ethiopia (39, 42). The finding was also consistent with study conducted in six-middle and low income countries (41). This could be due to the fact that mothers may receive adequate nutritional counseling during antenatal care visit which enhances their nutritional intake and reduces the risk of low birth weight. The other possible explanation for this might be, there will be a chance for monitoring of fetal wellbeing and timely intervention of feto-maternal problems when there is adequate ANC visit. Additionally, there is routine nutritional and medical advice, as well as the provision of iron folic acid supplementation which help in growth of the fetus.

A vital aspect for optimal pregnancy outcomes is family support, which includes assistance from family, the baby’s father, and general functional support (25). According to a Ghanaian study, the social support system related to the mother’s living situation during the pregnancy, women who lived with extended family during pregnancy had higher likelihood of giving birth to LBW than women who lived with intimate partner (7). But our study did not find any significant association between maternal living status during pregnancy and low birth weight.

## Limitation

When using this study finding, the following limitations should be taken in to account. The study was conducted at health-facilities. Therefore, the findings might not be generalizable to the total population. Again, the sample size used was also smaller, which might hinder the findings not nationally representative. Because some of information was recorded as reported by the women, the result might be affected by recall bias. Moreover, the maternal stress identified as the predictor of LBW was not studied at which gestational trimester and whether the stress is acute or chronic one. Again, the study did not include the environmental factors, particularly residential greenness, which has positive association with the birth outcome (43).

## Conclusion

The finding of this study revealed that, lack of nutritional counseling, maternal malnutrition (MUAC <23 cm), maternal stress during pregnancy and Antenatal follow up <4 visit were significant predictors of with low birth weight. Generally, almost all maternal risk factors identified in this study are modifiable risk factors. The study findings call upon the need of psychological support of the mothers during pregnancy, enhancing integrated nutritional counseling and intended frequency of ANC follow-up. Further study also is recommended to identify the causal pathway of maternal stress and the neonatal birth outcome in Ethiopia using clinical trial study.

## Data availability statement

The raw data supporting the conclusions of this article will be made available by the authors, without undue reservation.

## Ethics statement

The study was ethically approved by the Research Ethics and Review Committee of Wollega University with the reference number, WU/RD/534/2014. The studies were conducted in accordance with the local legislation and institutional requirements. Written informed consent for participation in this study was provided by the participants' legal guardians/next of kin.

## Author contributions

BRF, YM, and FF developed the concept and reviewed the literature. YM and BRF performed the data analysis. YM, BRF, BB, EA, and FF discussed the findings and proofread the manuscript for spelling and grammar. BRF wrote the first draft. BB and EA reviewed the manuscript. All authors contributed to the article and approved the submitted version.

## Abbreviations

ANC: Antenatal care
AOR: Adjusted Odds Ratio
COR: Crude Odds Ratio
MUAC: Mid-upper arm circumference
LBW: Low birth weight
EDHS: Ethiopian Demographic and Health Survey
SPSS: Statistical Power in Social Sciences
SD: Standard Deviation
STROBE: Strengthening the Reporting of Observational studies in Epidemiology
